# Supplementary material for: Temporal dynamics of the vaginal microbiome and host immune markers before, during, and after metronidazole treatment for bacterial vaginosis
Source: mSystems. 2025 Jul 3;10(7):e00380-25. doi: 10.1128/msystems.00380-25 (PMC12282074; doi:10.1128/msystems.00380-25)
Supplement: Fig. S1 — Bacterial abundances over time. [file msystems.00380-25-s0001.pdf]

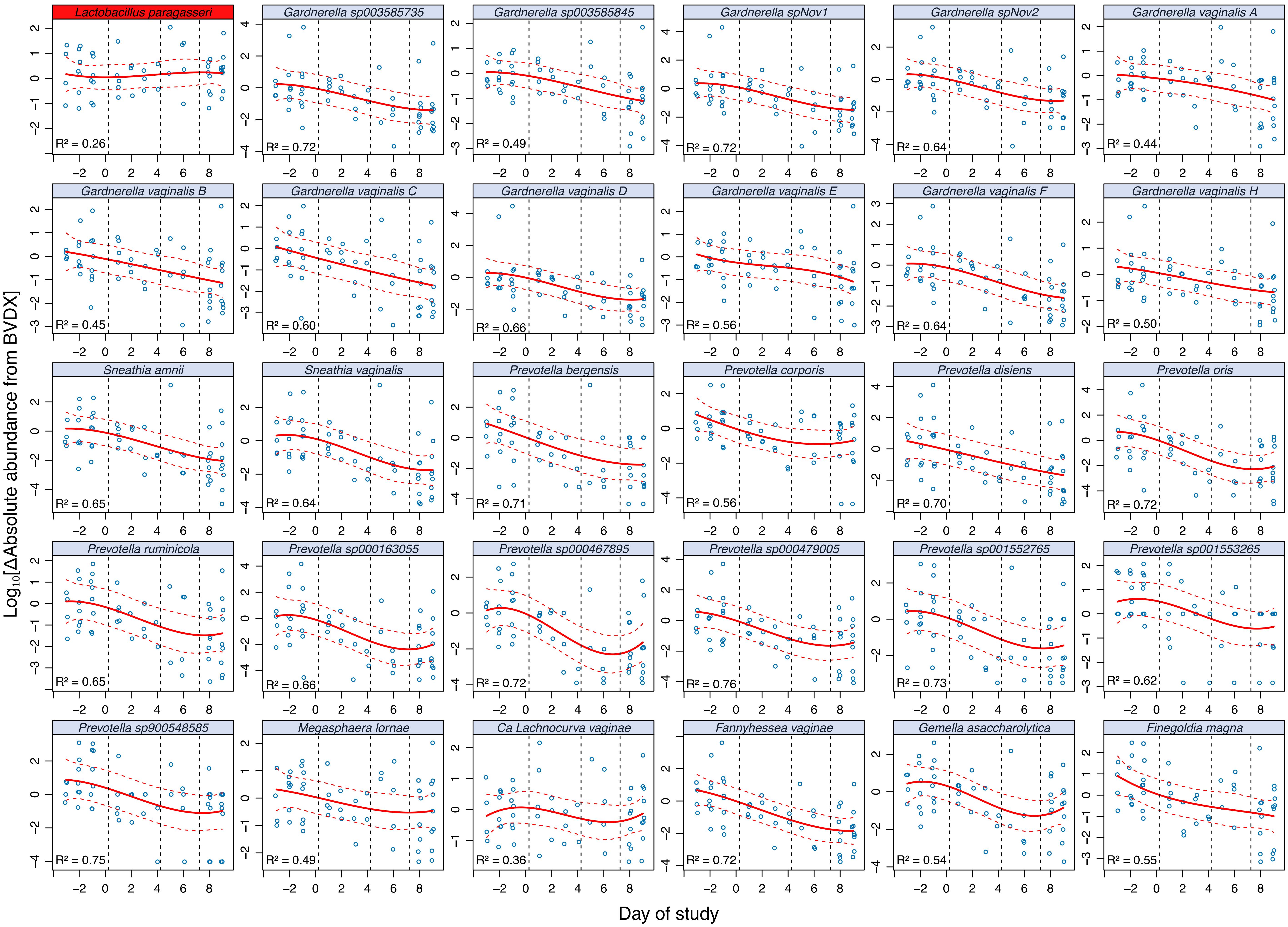

Figure S1. Bacterial abundances over time. The estimated absolute abundance (log10 transformed) of remaining taxa included in DL (red) and BVT (red) for all participants were plotted across all of the study days, with days relative to BVDX, which is represented by day 0. Negative days are those prior to SBV, and day 1 represents the first day the participant started their 7-day course of oral MET. Mixed effects models (solid red lines and 95% confidence intervals shown with dotted red lines) were generated for each species, with the study day as the fixed effect, estimated absolute abundance as the response variable, and participant ID considered a random effect. The conditional R2 values associated with the respective taxa are displayed.
